# Cannabis as entheogen: survey and interview data on the spiritual use of cannabis

**DOI:** 10.1186/s42238-020-00032-2

**Published:** 2020-09-22

**Authors:** Petter Grahl Johnstad

**Affiliations:** University of Bergen, Rosenbergsgate 39, 5020 Bergen, Norway

**Keywords:** Cannabis, Entheogens, LSD, Motivations for use, Psychedelics, Spirituality, Tolerance

## Abstract

**Background:**

While cannabis has a long history of spiritual use, its normalization in Western societies during the last decades has led to more recreational use. This study aimed to explore the characteristics of spiritual cannabis use as compared to recreational use and to the use of psychedelics such as LSD and psilocybin.

**Methods:**

The study employed a mixed methods research design that involved both qualitative interviews and a quantitative survey. Participants in interviews (*N* = 29) were recruited at various online fora for individual interviews via private messaging, and were queried in depth about their use of entheogens such as psilocybin, LSD, and DMT in spiritual contexts. The Cannabis and Psychedelics User Survey (CPUS) was constructed on the basis of the reports from these interviews, and recruited 319 participants (median age 33; 81% male) from seven different online communities. The online survey consisted of three main sections, with the first asking about demographics, personality, current and past affiliation to spiritual or religious traditions, and non-psychedelic drug use, and the second and third sections containing questions about motivations for, experience with, and consequences of cannabis and psychedelics use. The main statistical analyses used were multivariate linear and logistic regression analysis, which identified the effect from having a spiritual motivation for cannabis use on various aspects of the cannabis experience while controlling for a range of demographic, personality, and drug use variables.

**Results:**

Respondents differentiated clearly between the use of psychedelics and cannabis. Their use of the psychedelic drug they chose for the survey was restricted to a median of 1–10 use occasions per year, and 69% of participants endorsed having a spiritual motivation for use. Cannabis, on the other hand, was used a median of 51–100 times per year, and 25% of participants endorsed having a spiritual motivation for use. This minority of spiritual cannabis users differed significantly from non-spiritual users in how they approached cannabis use and in the type of experiences their use gave rise to. In multivariate logistic regression models, spiritual motivation was a significant predictor (*p* < .05) of experiences of insight, connectedness, joy, love, and unity with transcendent forces.

**Conclusions:**

The study found evidence of a group of spiritual cannabis users who tended to regard cannabis as an entheogen. These spiritual cannabis users had a different mode of engagement with cannabis than recreational users, and reported cannabis experiences that in some aspects resembled experiences with psychedelics. Recent research has not given much attention to spiritual aspects of cannabis use, but the study indicates that spiritually motivated use remains prevalent and deserves further study.

## Background

Cannabis has a long history of use in spiritual contexts (Fuller 2000). Archaeological evidence points to ritual cannabis use in China 2500 years ago (Jiang et al. 2006; Ren et al. 2019) and in Judahite worship in Israel dating back to the eighth century BCE (Arie et al. 2020). In India, spiritual cannabis use probably goes back to prehistoric times and has been associated with the worship of Śiva (Shiva) (Rätsch 2005; Russo 2005, 2007). One of Śiva’s epithets is ‘Lord of Bhang’, which refers to an edible preparation of cannabis, although devotees today more commonly smoke their ‘ganja’ (cannabis) in a ‘chillum’ (clay pipe) (Godlaski 2012). This tradition spread perhaps most notably to Jamaica, where Rastafarians smoke ganja – sometimes in a chillum, but more often as a ‘spliff’ (joint) – and may consider the act a sacrament (Chevannes 1994). During the 1960s, furthermore, cannabis use spread among Western countercultural movements, and often had spiritual overtones. According to Fuller (2000), the act of smoking cannabis during these years “was something like a rite of initiation into the religious underground,” whereby one joined “a community that valued nonconformity, peacefulness, and quiet introspection” (p. 145). One notable spokesperson for the age – the philosopher Alan Watts (1968) – endorsed cannabis as the ‘psychedelic’ that in his experience was best suited for moving into a state of ‘cosmic consciousness’, although he found that once the gate was opened, he was gradually able to move into this state without using drugs.

As cannabis use became normalized in Western societies during the 1970s and 1980s, however, it seems to have lost much of its spiritual connotations and became, in Fuller’s words, “just one more intoxicant alongside others” (Fuller 2000, p. 145). That normalization and de-spiritualization walked hand in hand here should probably not surprise us, at least if we follow Taves (2009) in identifying *specialness* as the central basis on which people deem certain things as belonging to the domain of religion or spirituality. If spirituality is about ‘things set apart’ and marked as special because of their anomalous nature, anything that is normalized can only have a tenuous connection to the spiritual.

The normalization of cannabis use is reflected in surveys of usage patterns. According to the 2019 World Drug Report (UNODC (United Nations Office on Drugs and Crime) 2019), the proportion of daily or near-daily cannabis users in the United States doubled during the years 2002–2017, while lifetime prevalence rates during the same period saw only a modest increase. The World Drug Report did not offer any estimates for cannabis usage patterns in other parts of the world; in Colorado, however, the report estimated that 27% of adult (age 21+) cannabis users had daily or near-daily use, while the median usage pattern remained more moderate at five use occasions per month. By contrast, Kumar et al. (2019) found a median use frequency of 250 days last year in a sample of 8345 US-resident respondents (median age = 23). In Europe, the European Monitoring Centre for Drugs and Drug Addiction estimated that while 27.4% of all adults in the European Union have tried cannabis during their lives, about 1% are current daily or near-daily users (EMCDDA (European Monitoring Centre for Drugs and Drug Addiction) 2019).

Writers who emphasize the spiritual potential of cannabis often warn against overuse. “With this plant you can free yourself and you can go into other realms of reality, transcendental realms of reality,” the Brazilian ayahuasquero Mariano da Silva (2017), p. 166 promised, but in order to obtain such ‘special effects’ you must have the discipline not to overindulge. For with overuse, it “starts to be common, almost normal. It loses quality” (p. 164). Gray (2017) similarly found that “engaging with the herb less frequently can make a big difference to the depth of a particular encounter” (p. 107). For these writers overuse implies trivialization, which must be avoided for the cannabis use to maintain its spiritual value. Gray also advised that the combination of cannabis use with some form of meditation practice tends to intensify its effects: “When you’re active and the thinking brain is engaged while under the influence, you may find the effects much milder than if you can sit still, avoid head traffic, and breathe into the space that cannabis opens up” (p. 74). While many users today seem to regard cannabis as a mild drug, there is no reason to assume that this applies to all. To take one notable example, Shulgin and Shulgin (1991, 1997), who invented and self-experimented with a great number of psychedelic tryptamines and phenethylamines, reported that cannabis was too intense for them (1997, pp. 48–63).

Previous research has identified five primary motivations for cannabis use: coping, enhancement, social, conformity, and expansion (Bresin and Mekawi 2019; Simons et al. 1998). In this model, coping refers to a wish to escape from problems, enhancement to a wish for pleasant feelings and excitement, the social motive is about increasing sociability, and conformity refers to a wish to use cannabis in order to fit in with the social group. Simons et al. (1998) added the expansion motive to account for desires to know oneself better, be creative and original, expand one’s awareness, and understand things differently. While none of these five motivations links directly to spirituality, both enhancement and expansion relate to factors that have previously been identified as important aspects of entheogenic spirituality (Johnstad 2018). Bresin and Mekawi (2019) performed a meta-analysis of the relations between cannabis use motives and outcomes, and found that coping motives predicted both a higher cannabis use frequency and more problematic use, while expansion and enhancement motives were associated with a higher frequency of use, but not with problematic use.

In this article, the term ‘psychedelics’ means the group of drugs named after the Greek words ψυχή (psyche), meaning soul or mind, and δηλείν (delein), to reveal or manifest. The classical psychedelics include mescaline (the active constituent of the cactus peyote), psilocybin (the active constituent of ‘magic mushrooms’), lysergic acid diethylamide (LSD) and N,N-dimethyltryptamine (DMT). Especially when used in spiritual contexts, psychedelics are also sometimes referred to as ‘entheogens’, which is derived from ἔνθεος (entheos), meaning inspired or filled with God, and γενέσθαι (genesthai), which means to come into being.

The article is based on an explorative mixed methods study involving both a quantitative survey and qualitative interviews. The study aimed to explore the characteristics of spiritual cannabis use as compared to both psychedelics use and what may be called recreational or non-spiritual cannabis use. As many cannabis users have both medical and recreational motivations for use, the study did not differentiate between medical and other forms of use; one item in the motivations battery of the survey allowed participants to indicate that they used cannabis for medical conditions. The basic hypothesis of the study was that most respondents would differentiate clearly between entheogens such as the classical psychedelics and recreational drugs such as cannabis and alcohol, but that a minority would regard cannabis as an entheogen. I expected to find significant differences between spiritual and recreational cannabis users in how they approached the drug in terms of motivation and usage pattern.

## Materials and methods

In the interview study, 29 current or past users of entheogenic drugs were interviewed about their experiences in two phases of study during the years 2015–2016 and 2019. Participants were recruited from a broad range of Internet communities, including norshroom.org, psychonaut.com, norcan.org, www.dmt-nexus.me, various Reddit groups, and actualized.org, either via general recruitment threads that explained the purpose of the study and invited people to participate, or via private messages to individual users who had previously posted to threads comparing cannabis and psychedelics. Criteria for selection were adulthood (18+) and current or past psychedelic use in self-identified spiritual contexts; these criteria were stated clearly in initial invitations, and no individuals expressing a wish to participate in the study were in fact excluded. Interviews were asynchronous and Internet-mediated, and participants were encouraged to interact with the interviewer via anonymized email or messaging that protected their identity from the researcher. Most interviews lasted from two to four weeks. In communications with interviewees, the term ‘spiritual’ was left undefined to avoid imposing limits on its content. This approach, inspired by Ammerman (2014), allowed for subsequent analysis of participants’ usage of the term, and such an analysis of entheogen users’ presentation of their spirituality is available in Johnstad (2018).

The study was designed in conformity with Norwegian Social Science Data Services ethical guidelines. Ethical approval for the first phase of the interview study was obtained from the Norwegian Social Science Data Services (NSSDS, reference 40,281/3/KH). Because privacy criteria were fulfilled, NSSDS waived ethical approval for the second part of the interview study, as well as for the survey. The study emphasized the preservation of participant anonymity, and aimed to ensure that no participant would be identifiable either to the researcher or to readers of published material. A few narratives have been translated from Norwegian, and statements have been edited for brevity and relevance. Insignificant details have sometimes been altered to preserve anonymity. Participants gave their informed consent to be included in the study, and were asked to read through and verify the use of their narratives. As interviews took the form of written communication (email or private messages at the forum), transcription was unnecessary. Data were analyzed using thematic analysis (Braun and Clarke 2006) and Kvale and Brinkmann’s (2015) procedure for meaning condensation, and themes were constructed in an open-ended, exploratory, and data-driven comparative analysis of participant narratives. The interview process allowed for the resolution of ambiguities through follow-up questions.

The Cannabis and Psychedelics User Survey was constructed on the basis of these interviews, with questions and the range of possible survey responses being based on themes identified in the interview analysis. In particular, the motivations for cannabis and psychedelics use and the characteristics of resulting experiences were based on information obtained from interviews. Before the survey was deployed, it went through a round of asynchronous testing on 18 volunteers recruited online, although this resulted only in minor revisions. The survey was made generally available online via SurveyXact from April to September 2019 for self-selected participation. It was fully anonymous and recorded no identifying participant information, including IP addresses. Several articles based on the Cannabis and Psychedelics User Survey are currently in preparation (Johnstad 2020a, 2020b; Johnstad PG: The psychedelic personality: personality structure and associations in a sample of psychedelics users, forthcoming). The survey text and the dataset are available as online attachments.

Participants for the survey were obtained from seven communities: www.shroomery.org, www.dmt-nexus.me, www.bluelight.org, the Facebook page for Portland Psychedelic Society, the Reddit group r/Psychedelics, the Norwegian Association for Safer Drug Policy, and an informal group of psychedelics users in Bergen, Norway. Participants were recruited either via invitation threads started at each forum or via a snowballing email invitation. Women were especially invited to participate in the survey. The only inclusion criteria were adulthood (18 years or older), the ability to understand English well, and having experience with a commonly used psychedelic drug. Individuals who did not meet the inclusion criteria were linked to a shorter version of the survey, and their data were not used in the analyses. Respondents reported using between 10 and 30 min to complete the survey.

### Measures

The Cannabis and Psychedelics User Survey included basic demographic questions relating to age, gender, education, work status, and relationship status. Gender was measured with three categories (female, male, and other), but when the gender variable was used as a control in statistical analyses, seven participants who indicated an “other” gender were excluded from the analysis. Education was quantified from 1 = “Have not completed high school” to 6 = “PhD”. Participants were also asked about their religious or spiritual background and their present religious or spiritual affiliations, as well as their current spiritual practice. Further questions examined their usage history and/or present use of cannabis and the psychedelic drugs of the 2C family (2C-B [2,5-dimethoxy-4-bromophenethylamine] etc.), 5-MeO-DMT (5-methoxy-*N,N*-dimethyltryptamine), Ayahuasca (or analogues), smoked DMT (*N,N*-Dimethyltryptamine), LSD (Lysergic acid diethylamide), MDMA (3,4-Methylenedioxy​-methamphetamine), Mescaline/Peyote, Psilocybin/Magic mushrooms, and *Salvia divinorum*. The survey asked participants to choose one psychedelic drug from this list that they had experience with, and they were queried about their motivations for the use of this drug and asked to characterize emotional, cognitive and relational aspects of their most meaningful experience with the drug, of a typical experience, and of their worst experience. This included an assessment of the meaningfulness of the experience taken from Griffiths et al. (2006), where participants rated the experience on a six-level scale (from 1 = “Most meaningful experience of your life” to 6 = “An everyday experience”). Finally, they were asked to characterize the consequences of their use of this drug for their physical health, psychological health, personal happiness, ability to get along with other people, and spiritual practice, each of which was measured on a five-level Likert scale (from 1 = “Serious worsening” or similar to 5 = “Serious improvement” or similar). The same range of questions were asked about cannabis for participants who had experience with this drug (95% of the sample). In addition, participants were asked to rate their current use of a range of non-psychedelic drugs quantified as 1 = “Daily”, 2 = “A few times per week”, 3 = “A few times per month”, 4 = “A few times per year”, and 5 = “Never”.

In order to measure the personality of the participants, the survey included a version of Gosling et al.'s (2003) Ten-Item Personality Inventory (TIPI), measured on a five-level Likert scale from “disagree strongly” to “agree strongly”. The TIPI is a concise measurement tool with only two items for each Big Five trait, but has been shown to have adequate construct validity, test–retest reliability, and patterns of external correlates (Gosling et al. 2003). TIPI scores were normalized for comparisons with available norms based on a seven-level scale according to the following formula: TIPI_normalized = ((TIPI_original – 1) * 6/4) + 1.

The survey also included a version of Nicholson et al. (2005) Risk Taking Index (RTI), measured on a five-level scale from “never” to “very often”. The original RTI contained an item for health risk that related to substance use, and to adapt the scale to a sample of cannabis and psychedelics users this item was removed. Thus, the modified RTI used for this survey included only five items: recreational risk, career risk, financial risk, safety risk, and social risk. To compensate for the removal of health risk in this population of psychedelics users, the combined overall RTI score was multiplied by 6/5. The original RTI asked participants to assess their risk taking both now and in the past, combining the two assessments into an overall score, while the modified RTI used in this survey, in order to preserve participants’ time, asked for only one assessment. Individual RTI scores for each risk domain were normalized for comparisons with available norms by multiplying the score by 2, thus in effect equalizing scores for the past and the present. As Nicholson et al. (2005) found that risk-taking decreases with age, the substitution of past scores with present scores in the modified RTI should serve to reduce risk taking scores as compared to the original RTI. As detailed in Johnstad PG: The psychedelic personality: personality structure and associations in a sample of psychedelics users, forthcoming, risk taking scores in the present study were, nevertheless, uniformly higher than the scores presented by Nicholson et al. (2005).

### Statistical analysis

In order to explore differences in motivations for drug use, characteristics of drug experiences, and self-assessed consequences of drug use, multivariate regression was used to assess the impact of spiritual motivation while controlling for commonly used demographic covariates (Hendricks et al. 2015; Nour et al. 2017) as well as the Big Five personality traits, the overall risk taking score (RTI), and the usage frequency of cannabis, psychedelics, and a range of non-psychedelic drugs. Separate multivariate logistic regression analyses were used to identify the independent variables that predicted dependent variables related to motivations for drug use and characteristics of drug experiences, and multivariate linear regression analyses were used to identify the independent variables that predicted dependent variables related to consequences of drug use. For each multivariate regression, independent variables were gender (coded as female = 0, male = 1), age, education, the six personality traits, five general drug use variables (coded from 1 = “Daily” to 5 = “Never”), two variables for cannabis and psychedelics use occasions the last 12 months (coded from 1 = “Zero” to 5 = “101+”), a variable for the duration of cannabis experience (coded from 1 = “Less than a year” to 5 = “10+ years”), and a dichotomous variable for whether or not the participant endorsed having a spiritual motivation for cannabis use (yes = 1). The multivariate linear regression analyses added a dichotomous variable for whether or not the participant endorsed having an escapist motivation for cannabis use (yes = 1). In all these analyses, ordinal variables were treated as continuous. Data was analyzed with IBM SPSS Statistics 25.

## Results – interview study

### Participant characteristics

Participants in the interview study were not always willing to provide demographic information. In order to reduce participation stress, only a minimum of such information was requested. Of the 22 participants who provided their gender and age, 20 were male and two female. The mean age was 35.6, with a range from the early 20s to the late 50s. Four were married (two with children), four were in stable relationships (one with children), six were single, and one in the middle of a break-up. Eleven held steady jobs in retailing, education, music teaching, journalism, industrial services, IT consulting, accounting, and as a hospital worker, one was a business owner, two were students, one was unemployed, and one used to work as a kindergarten assistant but was recently disabled because of an inherited condition.

### Usage pattern

In interviews with spiritually motivated cannabis users, there were two main trends for usage pattern. The first involved interviewees who were currently daily or near-daily users. For the most part, these people acknowledged that frequent use diminished their cannabis experiences, but maintained that this usage pattern was still of spiritual importance to them:

I have had many spiritual experiences with cannabis, and continue to use it for this purpose, although my overuse has dulled the experiences a bit. (ID11)

The other trend involved interviewees who were consciously limiting their usage frequency in order to maintain the spiritual value of their cannabis practice. Their usage pattern varied from about once per week to a few times per year:

My personal experience with cannabis has been very helpful. When I don’t use it for a week or two I’m getting very good trips. It feels like during my trip a part of my brain gets unlocked. (ID09)

Because it is very intense for me, I only do cannabis a few times every year. Also it’s my experience that if I do it too often it gets less intense, and therefore less meaningful for me. I want it to be a special, transformative, revelatory experience, and in order to give it the space it needs I must portion it out. (ID19)

### Motivations for use

Participants in interviews were asked about their motivations for starting to use entheogens, and sorted themselves into three different groups. The first group entered into the world of entheogens as part of an explicitly spiritual quest, choosing to engage with these drugs in order to obtain spiritual experiences. The second group expressed a general curiosity about the psychological effects that was not explicitly spiritual, but involved a wish to explore the realm of inner experience, and the third group was just socializing, partying, and having fun. Regardless of their initial motivation, all these interviewees eventually developed a spiritual motivation for continued entheogen use.

I experimented with cannabis because I was curious about it. The first five times or so – this was over a period of maybe six or seven years – it didn’t do anything for me. Then suddenly my world exploded with spiritual revelation. (ID19)

I have searched for a religious/mystical experience since I was a boy. I came across information about LSD, the Gospel of Thomas, and the Tibetan Book of the Dead on the Internet when the rest of my class in high school was on a trip abroad. (ID30)

### Characteristics of drug-induced experiences

Interviewees described entheogenic experiences as being characterized by insight into self, relations, and world, inner visions, feelings of peace, joy, and love, and occasional peak experiences involving ego dissolution and what was interpreted as contact with transcendent forces. The majority did not count cannabis as an entheogen, however, and described the cannabis state as one of peaceful relaxation. Those who did regard cannabis as an entheogen usually – but not always – saw it as less intense than the classical psychedelics. Interviewees who valued the spiritual dimension of cannabis often had a meditative or introspective approach to it.

I found that moderate cannabis use very useful in maintaining a relaxed and meditative state of mind. I found that being in such a state most of the time meant that my conscious mind had a more efficient connection to my sub-conscious mind, which I believe to be the incarnate link between the consciousness of the animal and the Spirit that dwells ‘within’. (ID25)

Cannabis changed my life. It brought me into contact with something larger than life – a spiritual dimension to my existence. It made me realize what I now regard as fact: that there is much more to our human existence than we are usually aware of. This earthly life is only a small part of our true life, and to die from this world is only to return home. (ID19)

Cannabis is definitely psychedelic for me and expands my consciousness, but most times when I smoke my mind also gets quite foggy. Clear and discerning thinking is not as possible like on other psychedelics, therefore the others are far superior for me. (ID12)

### Consequences of drug-induced experiences

When asked to describe the long-term consequences of their entheogen use, participants in interviews pointed especially to psychological healing and personal growth. They claimed that entheogens had helped them deal with existential issues, personal problems such as social anxiety, and medical conditions such as depression, post-traumatic stress disorder, and addiction to alcohol, nicotine, and gambling. Different interviewees usually emphasized different entheogens as being especially helpful to them, but there were no discernable trends that differentiated cannabis from other entheogens.

Entheogens have helped me to see the wonder in life, and as a by-product, I have felt renewed energy in my studies at my university and my overall attitude. I feel very grateful for my family and all of those close to me. Life is good! (ID31)

Entheogens helped me realize the importance of letting go rather than clinging on to anger or grief. Another thing is that ‘I am my own responsibility’ and therefore have to take ownership of my own emotions, plans for the future, economy, relations, etc. (ID23)

Marijuana seems to open up a part of my mind which seems to be able to think higher, better, and more lovingly than without it. Some of my biggest and most successful changes made in my small business have been thought up while under the influence of marijuana. I have also healed a ton of my anxiety, depression, and social anxiety with marijuana. (ID11)

Asked about negative consequences, interviewees emphasized the dangers of overuse. Because cannabis and 3,4-Methylenedioxymethamphetamine (MDMA) were seen as giving rise to less intense experiences than other entheogens, they were regarded as easier to overuse. Participants also pointed to a tolerance effect where over-frequent use reduced the intensity of the experience.

If you overdo it, you will have less and less interesting experiences. (ID30)

I cannot abuse mushrooms in the same way as cannabis. In a way I get filled up by a mushroom trip. Cannabis is not as intense an experience. (ID26)

## Results – quantitative survey

### Participant characteristics

A total of 527 forms were submitted, but 202 of these were empty or near-empty and were excluded from analysis. Six responses with substantial discrepancies on repeated drug use assessments were also excluded. Of the 319 included participants, 213 completed the full survey, while 106 opted out from parts of it. There were a number of differences between the two groups: among other things, participants who completed the study had higher education (*t* = 2.68, *df* = 317, *p* = .008), were more likely to be a pensioner (*t* = 3.06, *df* = 212, *p* = .003), and had higher scores on the personality traits openness (*t* = 2.68, *df* = 109, *p* = .009) and conscientiousness (*t* = 2.00, *df* = 287, *p* = .047). See Additional file 1: Table A in the online appendix for a more comprehensive overview. It should be noted that these are uncorrected figures in a study with more than 300 variables, where 15 false positives might be expected with a 95% significance level. The two groups were not different in terms of having a spiritual motivation for cannabis use (*t* = 0.73, *df* = 263, *p* = .466), which was the main explanatory variable used in this study. Respondents were free to choose which psychedelic drug they would describe their interaction with in the survey, but usually chose a drug they had much experience with relative to other psychedelic drugs. In paired *t*-tests, the mean number of use occasions for their chosen drug significantly exceeded that of all other psychedelic drugs at *p* < .001.

An overview of participant characteristics for the survey, grouped according to whether or not they endorsed having a spiritual motivation for cannabis use, is provided is Table 1. The median participant was a male aged 32 with some university education, unmarried and childless but with a partner, situated in North America and working a full time job. Most participants reported having a religious background and a present religious or spiritual affiliation. However, there were substantial demographic differences between the spiritual and nonspiritual groups.

**Table 1:** Participant characteristics for 265 Internet survey respondents (grouped according to motivation for cannabis use)^a^

	Spiritually motivated users (*N* = 67)	Non-spiritually motivated users (*N* = 198)	Diff.
Age	12% 18–19 years	7% 18–19 years	*p* = .06
	36% 20–29 years	29% 20–29 years	
	28% 30–39 years	33% 30–39 years	
	16% 40–49 years	15% 40–49 years	
	3% 50–59 years	11% 50–59 years	
	5% 60+ years	5% 60+ years	
	(Median = 30, M = 32.6, SD = 11.5)	(Median = 34, M = 35.6, SD = 12.1)	
Gender	12% female, 85% male, 3% other	20% female, 78% male, 2% other	*p* = .16^c^
Relationship status	52% single	40% single	*p* = .08
	25% partner	32% partner	*p* = .32
	22% married	28% married	*p* = .39
	0% widow (er)	1% widow (er)	*p* = .56
Number of children	75% none	66% none	*p* = .15
	10% one child	12% one child	
	13% two children	14% two children	
	2% three or more children	8% three or more children	
	(M = .42, SD = .78)	(M = .63, SD = .99)	
Education	8% PhD	5% PhD	*p* = .96
	15% Master’s degree	15% Master’s degree	
	21% Bachelor’s degree	22% Bachelor’s degree	
	33% some university	38% some university	
	19% high school	16% high school	
	5% not completed high school	5% not completed high school	
	(M = 5.16 years, SD = 2.56 years)	(M = 5.03 years, SD = 2.32 years)	
Religious background^b^	15% Buddhist	9% Buddhist	*p* = .18
	21% Christian	24% Christian	*p* = .63
	8% Hindu	2% Hindu	***p*** **= .03**
	6% Jewish	2% Jewish	***p*** **= .05**
	3% Muslim	2% Muslim	*p* = .45
	22% New Age/Alternative	14% New Age/Alternative	*p* = .09
	34% Secular/Humanist	36% Secular/Humanist	*p* = .82
	48% other	41% other	*p* = .33
Religious affiliation at present^b^	51% Buddhist	23% Buddhist	***p*** **< .01**
	28% Christian	14% Christian	***p*** **= .01**
	30% Hindu	7% Hindu	***p*** **< .01**
	9% Jewish	3% Jewish	***p*** **= .04**
	8% Muslim	2% Muslim	***p*** **= .01**
	45% New Age/Alternative	22% New Age/Alternative	***p*** **< .01**
	34% Secular/Humanist	37% Secular/Humanist	*p* = .68
	42% other	47% other	*p* = .46
Occupation^b^	51% full time job	59% full time job	*p* = .26
	16% part time job	17% part time job	*p* = .89
	27% student	17% student	*p* = .07
	3% pensioner	4% pensioner	*p* = .83
	5% unemployed	5% unemployed	*p* = .85
	13% other	16% other	*p* = .66
Geographical location at present	60% North America	55% North America	*p* = .46
	24% Western Europe	30% Western Europe	*p* = .35
	6% Eastern Europe	4% Eastern Europe	*p* = .39
	5% Oceania	8% Oceania	*p* = .32
	3% Middle East	1% Middle East	*p* = .10
	2% South America	2% South America	*p* = .78
	2% Africa	1% Africa	*p* = .42
	0% Asia	1% Asia	*p* = .41
Personality traits	3.96 Extraversion	3.82 Extraversion	*p* = .54
	4.69 Conscientiousness	4.98 Conscientiousness	*p* = .19
	6.07 Openness	5.80 Openness	*p* = .08
	4.96 Agreeableness	4.73 Agreeableness	*p* = .20
	4.87 Emotional stability	4.79 Emotional stability	*p* = .71
	37.33 Risk taking	34.19 Risk taking	***p*** **< .01**
Years of cannabis experience	3.0% Less than a year	8.6% Less than a year	*p* = .06
	11.9% 1–3 years	18.2% 1–3 years	
	11.9% 3–5 years	11.1% 3–5 years	
	22.4% 5–10 years	21.2% 5–10 years	
	50.7% 10+ years	40.9% 10+ years	
	(Median = 10+ years)	(Median = 7 years)	
Cannabis use last 12 months	Median = 91 use occasions	Median = 67 use occasions	*p* = .31
Psychedelics use last 12 months^d^	Median = 3 use occasions	Median = 3 use occasions	*p* = .43
Alcohol use	14.9% Daily	3.5% Daily	*p* = .18
	17.9% A few times per week	18.7% A few times per week	
	25.4% A few times per month	29.8% A few times per month	
	23.9% A few times per year	31.8% A few times per year	
	17.9% Never	16.2% Never	
Amphetamine use	1.5% Daily	5.6% Daily	*p* = .65
	1.5% A few times per week	2.5% A few times per week	
	7.5% A few times per month	5.1% A few times per month	
	16.4% A few times per year	15.7% A few times per year	
	73.1% Never	71.2% Never	
Cigarette/tobacco use	32.8% Daily	31.8% Daily	*p* = .57
	6.0% A few times per week	5.1% A few times per week	
	9.0% A few times per month	6.1% A few times per month	
	10.4% A few times per year	10.1% A few times per year	
	41.8% Never	47.0% Never	
Cocaine use	0% Daily	0% Daily	*p* = .70
	1.5% A few times per week	0.5% A few times per week	
	4.5% A few times per month	1.5% A few times per month	
	14.9% A few times per year	22.2% A few times per year	
	79.1% Never	75.8% Never	
Opiate use	3.0% Daily	4.5% Daily	*p* = .20
	1.5% A few times per week	1.5% A few times per week	
	1.5% A few times per month	2.0% A few times per month	
	22.4% A few times per year	11.6% A few times per year	
	71.6% Never	80.3% Never	

Note: The ‘Diff.’ column indicates significant difference between the two groups on the Mann-Whitney *U* test, with significant values indicated in bold (p < = .05). ^a^Sums may differ from 100% because of rounding. ^b^Sums to more than 100% because respondents could choose several alternatives. ^c^Other gender (*N* = 7) excluded. ^d^This refers to the use of the psychedelic drug that participants chose to describe their interaction with in the survey. M = mean. SD = standard deviation

### Usage pattern

**Fig. 1:**
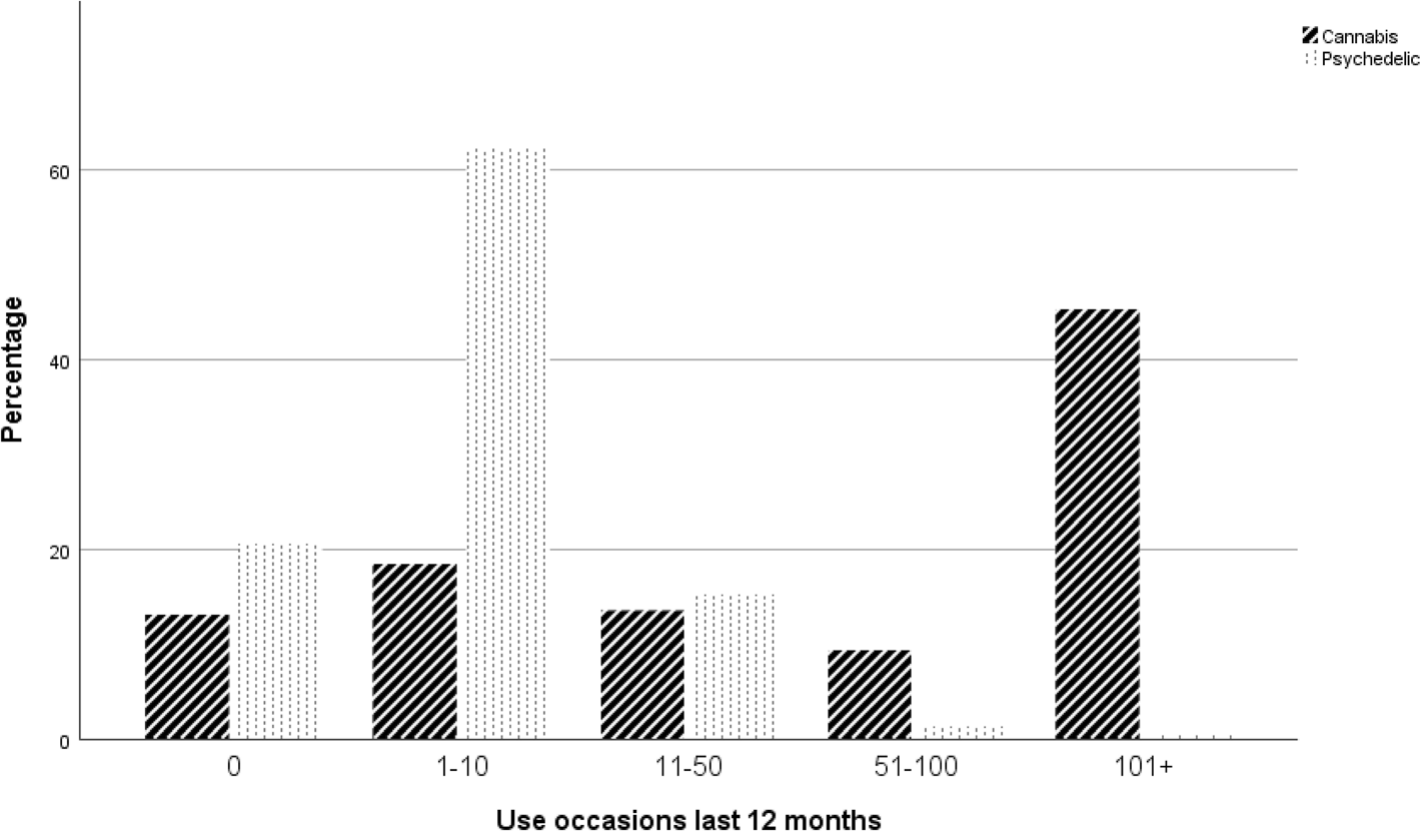
Cannabis and psychedelics use over the prior 12 months by 265 Internet survey respondents. Participants endorsed one of nine possible answers to the question “How often have you used [this drug] over the last 12 months?” for psychedelics (*N* = 228) and cannabis (*N* = 265). The nine original categories were combined into 5 to simplify the presentation

Participants reported substantial differences in their usage patterns for cannabis and psychedelics (Fig. 1). They reported a median of 1–10 use occasions for their chosen psychedelic and 51–100 use occasions of cannabis over the last 12 months, with a large minority (45%) reporting 101+ cannabis use occasions. Participants who endorsed having a spiritual motivation for cannabis use did not differ significantly in usage frequency from other participants (*t* = 1.43, *df* = 123, *p* = .155).

### Motivations for use

Participants reported significant differences in their motivations for cannabis and psychedelics use (Table 2). For cannabis, fun/party/recreation and socializing were the most commonly endorsed motivations, whereas the most endorsed items for psychedelics use related to self-exploration and personal growth. The subset of spiritually motivated cannabis users diverged substantially from the overall cannabis trend, however. These participants endorsed having motivations for cannabis use that often resembled those given for psychedelics use, with substantial majorities endorsing self-exploration, personal growth, and adventure as a motivation for cannabis use.

**Table 2:** Motivations for continued cannabis and psychedelics use among 265 Internet survey respondents

	Psychedelics (*N* = 228)		Cannabis (*N* = 265)		Spiritually motivated cannabis use (*N* = 67)
Adventure	54%	***	31%	***	69%
Curiosity	44%	***	22%	***	40%
Ego death experience	43%	***	5%	**	15%
Fun/party/recreation	41%	***	67%		72%
Insight and understanding for personal growth	84%	***	37%	***	76%
Psychological self-exploration	84%	***	37%	***	79%
Socializing	23%	***	56%	**	70%
Spiritual experience	69%	***	25%	(n/a)	(100%)
To cure or heal medical conditions	21%	*	30%		33%
To cure or heal personal problems	44%	***	26%	***	46%
To forget or escape from personal problems	8%	***	32%		37%

Note: The left column of stars indicates significant difference on the paired *t*-test between psychedelics and cannabis use (*N* = 219); the right column indicates significant difference on the independent *t*-test between spiritually motivated cannabis users (*N* = 67) and other cannabis users (*N* = 198): * p < = .05, ** p < = .01, *** p < = .001

Further statistical analysis focused on the differences between spiritual and non-spiritual cannabis use. Multivariate logistic regression analyses supported most of the differences identified in Table 2 between spiritual and non-spiritual cannabis use, as a spiritual motivation for cannabis use significantly predicted having adventure, curiosity, insight and understanding for personal growth, psychological self-exploration, and to cure or heal personal problems as additional motivations in regression models that controlled for age, gender, education level, personality traits, and drug use (Table 3). The dichotomous spiritual motivation variable that differentiated between the two types of cannabis use in Table 2 thus maintained its effect in multivariate regression models, which indicates that its effect is independent from a broad range of potential confounders related to demographics, personality, and drug use. In these models, the personality traits Agreeableness and Emotional stability, when controlled for the other variables in the model, positively predicted a search for adventure, while Openness positively predicted a wish for insight and understanding for personal growth. Emotional stability negatively predicted wanting to cure or heal personal problems.

**Table 3:** Motivations for cannabis use among 214 Internet survey respondents in multivariate logistic regression models

	Adventure			Curiosity			Insight and understanding for personal growth			Psychological self-exploration			To cure or heal personal problems		
	B	SE	*p*	B	SE	*p*	B	SE	*p*	B	SE	*p*	B	SE	*P*
*Intercept*	-5.69	2.99		-1.17	2.89		**-13.96**	**3.31**	***	-3.49	2.80		-.63	3.00	
Age	-.23	.19		.03	.18		-.24	.18		-.25	.17		-.34	.20	
Gender (M)	-.11	.52		.00	.51		-.34	.47		-.22	.48		-.62	.49	
Education	-.03	.16		.05	.16		**.48**	**.18**	**	.06	.16		-.14	.16	
Extraversion	-.15	.13		.00	.14		.04	.13		-.11	.12		.04	.13	
Conscientiousness	.09	.16		.08	.16		.22	.15		.06	.15		.07	.15	
Openness	.26	.22		.11	.22		**.49**	**.22**	*	.29	.20		.24	.22	
Agreeableness	**.35**	**.15**	*	.01	.15		.17	.15		.26	.14		.02	.16	
Emotional stability	**.41**	**.16**	**	.27	.15		.14	.14		-.02	.14		**-.36**	**.15**	*
Risk taking	.01	.03		.03	.03		.03	.03		.02	.02		.04	.03	
Alcohol use	.20	.17		.02	.17		.25	.17		.00	.16		-.07	.17	
Amphetamine use	-.08	.19		**-.47**	**.18**	*	-.23	.20		-.14	.18		-.07	.20	
Cigarette/tobacco use	-.10	.12		.17	.12		.15	.12		-.01	.11		.00	.11	
Cocaine use	.17	.41		-.16	.41		.65	.42		-.17	.41		.01	.40	
Opiate use	-.19	.20		-.19	.22		.09	.20		.24	.21		-.23	.20	
Psychedelics use 12 months	-.12	.10		-.09	.10		-.06	.10		-.04	.10		.06	.10	
Years of cannabis experience	.01	.18		**-.35**	**.18**	*	.23	.18		.02	.16		.05	.19	
Cannabis use 12 months	.00	.07		.06	.07		**.17**	**.07**	*	.02	.07		.14	.08	
Spiritual motivation	**2.49**	**.43**	***	**1.32**	**.41**	***	**2.37**	**.44**	***	**2.56**	**.42**	***	**1.30**	**.41**	***

Note: *N* = 214. Results from multivariate logistic regression models. Each model contains 18 independent variables: age, gender (coded as male = 1), education (quantified from 1 = “Have not completed high school” to 6 = “PhD”), the Big Five personality traits, the overall Risk Taking score (RTI), five variables for current drug use (quantified from 1 = “Daily” to 5 = “Never”), two variables for the number of use occasions for psychedelics and cannabis over the last 12 months, a variable for the number of years of cannabis experience (quantified from 1 = “Less than a year” to 5 = “10+ years”),and a dichotomous variable for spiritual motivation. Results from five models are shown, one for each of five dependent variables: adventure (model Nagelkerke R-square = .41), curiosity (model Nagelkerke R-square = .21), personal growth (model Nagelkerke R-square = .44), self-exploration (model Nagelkerke R-square = .38), and healing personal problems (model Nagelkerke R-square = .27). Values in bold represent statistically significant associations. B = unstandardized regression coefficient, SE = standard error, * *p* < = .05, ** *p* < = .01, *** *p* < = .001

### Characteristics of drug-induced experiences

Participants were asked to characterize emotional, cognitive and relational aspects of a typical experience with cannabis and a psychedelic drug. For most characteristics, they reported significant differences between psychedelic and cannabis experiences (Table 4). The discrepancy was particularly large for characteristics indicating a mystical-type experience, such as ego death, ineffability, and contact or unity with transcendent forces, but was also substantial for more mundane characteristics involving insight and emotions such as joy, love, sadness, surprise, and fear. In sum, these differences in levels of endorsement seem to indicate that participants regarded experiences with psychedelics as more noteworthy and ‘special’ than experiences with cannabis. Spiritually motivated cannabis users endorsed characteristics relating to insight and positive emotions at higher levels than other cannabis users, however. For these users, the cannabis experience, at least in certain respects, tended to resemble a psychedelic experience.

**Table 4:** Comparisons of drug experience characteristics among 250 Internet survey respondents

	Typical psychedelic experience (*N* = 220)		Typical cannabis experience (*N* = 250)		Spiritually motivated cannabis experience (*N* = 66)
Anger or hate	2%		1%		2%
Confusion	24%		21%		21%
Contact with non-ordinary beings	25%	***	3%		3%
Contact with transcendent forces	34%	***	5%		11%
Disgust	5%	*	2%		5%
Ego death or dissolution	33%	***	4%		9%
Fear	24%	**	14%		17%
Feeling of homecoming or return to your essence	60%	***	27%	***	49%
Feeling of isolation from other people	12%		17%		15%
Improved connection with nature	75%	***	48%	***	73%
Improved connection with other people	67%	***	44%	***	64%
Inner visions	57%	***	14%	**	27%
Insight into the world	78%	***	38%	***	56%
Insight into your relations	74%	***	42%	***	65%
Insight into yourself	86%	***	51%	**	65%
Joy	84%	***	56%	**	71%
Love	76%	***	37%	***	58%
Peace	82%		72%		77%
Regrettable behavior towards others	6%		4%		3%
Sadness	19%	***	6%		11%
Surprise	42%	***	8%		14%
Unity with transcendent forces	41%	***	4%	*	12%
Words cannot describe the experience	49%	***	7%		12%

Note: The left column of stars indicates significant difference on the paired *t*-test between a typical psychedelic and cannabis experience (*N* = 212); the right column indicates significant difference on the independent *t*-test between spiritually motivated cannabis users (*N* = 66) and other cannabis users (*N* = 184) for a typical cannabis experience: * p < = .05, ** p < = .01, *** p < = .001

The spiritual motivation variable retained its impact in multivariate logistic regression models that controlled for a range of demographic, personality, and drug use variables. In these models, having a spiritual motivation for cannabis use significantly predicted most of the characteristics in Table 4 that distinguished spiritual and recreational experiences (Table 5). This indicates that the effect from spiritual motivation was independent from demographic, personality, and drug use differences between the participants. In addition to the strongly significant spiritual motivation variable in these models, the personality trait Conscientiousness positively predicted experiences of connectedness to nature and to other people, as well as experiences of feeling love. A higher frequency of cannabis during the last 12 months also predicted experiences of connectedness and love, which may reflect that respondents who obtained such effects from cannabis use were encouraged to repeat the experience more often. Finally, higher scores on risk taking predicted experiences of connectedness with nature, perhaps indicating that high risk takers were more likely to use cannabis outdoors.

**Table 5:** Spiritual motivation as predictor of cannabis experience among 214 Internet survey respondents in multivariate logistic regression

	Feeling of homecoming or return to your essence			Improved connection with nature			Improved connection with other people			Insight into the world			Insight into your relations			Love		
	B	SE	*p*	B	SE	*p*	B	SE	*p*	B	SE	*p*	B	SE	*p*	B	SE	*p*
*Intercept*	-1.65	2.69		**-5.27**	**2.64**	*	-3.07	2.46		1.27	2.50		-2.50	2.43		-2.98	2.62	
Age	.02	.17		.10	.16		-.17	.15		.22	.15		-.06	.15		.02	.16	
Gender (M)	-.49	.45		-.17	.44		-.63	.44		-.80	.45		-.61	.42		-.46	.45	
Education	-.16	.15		-.01	.15		-.08	.14		-.14	.14		-.12	.14		**-.34**	**.15**	*
Extraversion	.06	.12		.11	.11		.21	.11		.11	.12		.01	.11		-.02	.12	
Conscientiousness	-.06	.14		**.34**	**.14**	*	**.38**	**.13**	**	.11	.13		.07	.13		**.37**	**.14**	**
Openness	.23	.19		.28	.18		-.22	.17		-.01	.17		.09	.17		-.08	.18	
Agreeableness	-.03	.14		.04	.13		-.12	.13		-.19	.13		.04	.13		.15	.14	
Emotional stability	.05	.13		-.01	.13		.21	.12		.21	.13		.11	.12		-.06	.13	
Risk taking	-.01	.02		**.04**	**.02**	*	.04	.02		.01	.02		.02	.02		.02	.02	
Alcohol use	-.06	.15		-.14	.15		.13	.14		.15	.15		.28	.14		.11	.15	
Amphetamine use	-.13	.18		-.20	.18		-.08	.18		-.12	.18		-.09	.17		.24	.20	
Cigarette/tobacco use	-.13	.10		.05	.10		.11	.10		.05	.10		.09	.09		.10	.10	
Cocaine use	.23	.37		.15	.37		.09	.35		**-1.04**	**.38**	**	-.27	.35		-.07	.37	
Opiate use	-.02	.19		-.17	.19		-.11	.18		.28	.19		.22	.18		-.20	.19	
Psychedelics use 12 months	-.07	.09		-.04	.09		-.03	.09		-.07	.09		-.09	.08		-.08	.09	
Years of cannabis experience	.10	.17		-.14	.15		.02	.15		.19	.15		**.28**	**.14**	*	.09	.15	
Cannabis use 12 months	.10	.07		**.24**	**.07**	***	**.15**	**.06**	*	.11	.06		.03	.06		**.17**	**.07**	*
Spiritual motivation	**1.19**	**.37**	***	**1.59**	**.41**	***	**1.18**	**.38**	**	**1.27**	**.38**	***	**1.24**	**.37**	***	**1.28**	**.38**	***

Note: *N* = 214. Results from multivariate logistic regression models. Each model contains 18 independent variables: age, gender (coded as male = 1), education (quantified from 1 = “Have not completed high school” to 6 = “PhD”), the Big Five personality traits, the overall Risk Taking score (RTI), five variables for current drug use (quantified from 1 = “Daily” to 5 = “Never”), two variables for the number of use occasions for psychedelics and cannabis over the last 12 months, a variable for the number of years of cannabis experience (quantified from 1 = “Less than a year” to 5 = “10+ years”), and a dichotomous variable for spiritual motivation. Results from six models are shown, one for each of six dependent variables: homecoming (model Nagelkerke R-square = .16), connection with nature (model Nagelkerke R-square = .32), connection with other people (model Nagelkerke R-square = .23), insight into the world (model Nagelkerke R-square = .27), insight into your relations (model Nagelkerke R-square = .19), and love (model Nagelkerke R-square = .27). Values in bold represent statistically significant associations. B = unstandardized regression coefficient, SE = standard error, * *p* <= .05, ** *p* <= .01, *** *p* <= .001

### Consequences of drug-induced experiences

Participants rated the consequences of their drug use as neutral or positive on all indicators, with significantly higher scores for psychedelics than for cannabis (Table 6). Spiritually motivated cannabis users rated the consequences of such use for their psychological health and spiritual practice significantly higher than the rest of the sample.

**Table 6:** Consequences of cannabis and psychedelics use among 225 Internet survey respondents

	Psychedelics (*N* = 213)		Cannabis (*N* = 225)		Spiritually motivated cannabis use (*N* = 60)
Physical health	3*.*66	***	3*.*23		3*.*37
Psychological health	4*.*26	***	3*.*32	*	3*.*55
Spiritual practice	3.89	***	3*.*42	***	3*.*82
Ability to get along with people	4*.*03	***	3*.*40		3*.*50
Personal happiness	4*.*27	***	3*.*54		3*.*67

Note: Numbers indicate average scores on a five-level Likert scale (range: 1–5). The left column of stars indicates significant difference on the paired *t*-test between psychedelics and cannabis use (*N* = 205); the right column indicates significant difference on the independent *t*-test between spiritually motivated cannabis users (*N* = 60) and other cannabis users (*N* = 165): * p < = .05, ** p < = .01, *** p < = .001.

In order to control the figures for cannabis use for the effects from possibly confounding variables, multiple linear regression analyses were performed using the five-level Likert scales as dependent variables (Tables 7). The analyses show that having a spiritual motivation predicted positive consequences for self-reported psychological health and spiritual practice when controlled for age, gender, education level, personality traits, and drug use. Having an escapist motivation, conversely, predicted negative consequences on every outcome except physical health and spiritual practice. The personality trait Conscientiousness predicted positive cannabis consequences across the board, as did having a higher frequency of cannabis use during the last 12 months. The latter finding is open to several interpretations, one of which might be that cannabis users who experience their use as beneficial will tend to increase the frequency of use. Lower opiate use predicted worse self-reported consequences of cannabis use, which may reflect opiate users comparing the consequences of cannabis use with the presumably more problematic consequences of opiate use, and as a result reporting favorably on the behalf of cannabis.

**Table 7:** Consequences of cannabis use among 200 Internet survey respondents in linear multivariate regression models

	Physical health			Psychological health			Spiritual practice			Ability to get along with people			Personal happiness		
	B	SE	*p*	B	SE	*p*	B	SE	*p*	B	SE	*p*	B	SE	*p*
*Intercept*	**2.70**	**.88**	**	**2.72**	**.94**	**	**3.11**	**.89**	***	1.69	1.03		1.55	.96	
Age	-.02	.05		.04	.06		**-.11**	**.05**	*	-.09	.06		-.07	.06	
Gender (M)	**-.32**	**.15**	*	**-.35**	**.16**	*	.00	.15		-.04	.17		.03	.16	
Education	-.01	.05		.07	.05		.01	.05		.02	.06		.04	.05	
Extraversion	.04	.04		.02	.04		.01	.04		.03	.04		.07	.04	
Conscientiousness	**.15**	**.05**	***	**.12**	**.05**	**	**.14**	**.05**	**	**.13**	**.05**	*	**.13**	**.05**	**
Openness	.01	.06		.12	.06		.05	.06		.02	.07		**.14**	**.06**	*
Agreeableness	.02	.04		.02	.05		-.07	.04		.03	.05		-.02	.05	
Emotional stability	.05	.04		.06	.05		-.00	.05		-.02	.05		.04	.05	
Risk taking	.01	.01		.01	.01		.01	.01		.01	.01		.01	.01	
Alcohol use	-.06	.05		-.04	.05		-.03	.05		-.04	.06		-.08	.05	
Amphetamine use	-.05	.06		-.04	.06		-.01	.06		-.02	.07		-.09	.07	
Cigarette/tobacco use	.02	.03		.01	.04		.01	.03		.04	.04		.07	.04	
Cocaine use	-.02	.12		-.05	.13		.04	.13		.22	.15		.06	.14	
Opiate use	-.08	.06		**-.23**	**.07**	***	**-.14**	**.07**	*	**-.18**	**.06**	*	**-.19**	**.07**	**
Psychedelics use 12 months	-.02	.03		-.03	.03		-.03	.03		.01	.04		-.01	.03	
Years of cannabis experience	.01	.05		-.03	.05		.00	.05		.04	.06		.02	.05	
Cannabis use 12 months	**.06**	**.02**	**	**.11**	**.02**	***	**.05**	**.02**	*	**.08**	**.02**	**	**.11**	**.02**	***
Escapist motivation	-.10	.13		**-.37**	**.14**	**	-.14	.13		**-.37**	**.15**	*	**-.34**	**.14**	*
Spiritual motivation	.19	.13		**.30**	**.13**	*	**.51**	**.13**	***	.07	.15		.07	.14	

Note: *N* = 200. Results from linear multivariate regression models. Each model contains 19 independent variables: age, gender (coded as male = 1), education (quantified from 1 = “Have not completed high school” to 6 = “PhD”), the Big Five personality traits, the overall Risk Taking score (RTI), five variables for current drug use (quantified from 1 = “Daily” to 5 = “Never”), two variables for the number of use occasions for psychedelics and cannabis over the last 12 months, a variable for the number of years of cannabis experience (quantified from 1 = “Less than a year” to 5 = “10+ years”), a dichotomous variable for escapist motivation, and a dichotomous variable for spiritual motivation. Results from five models are shown, one for each of five dependent variables: physical health (model adjusted R-square = .12), psychological health (model adjusted R-square = .26), spiritual practice (model adjusted R-square = .14), sociability (model adjusted R-square = .11), and personal happiness (model adjusted R-square = .24). Values in bold represent statistically significant associations. B = unstandardized regression coefficient, SE = standard error, * *p* <= .05, ** *p* <= .01, *** *p* <= .001

## Discussion

In this study, it was clear that cannabis means different things to different people. Many of the participants in the study drew a clear line between cannabis and psychedelics in terms of both their motivations for use and the characteristics of experiences. While often attributing spiritual and self-developmental characteristics to their psychedelics use, which was limited to a median of 1–10 use occasions per year for their chosen psychedelic drug, they regarded cannabis as a drug that could be used quite frequently for the more mundane purposes of recreation and relaxation. In the terms of Simons et al.’s (1998) model for cannabis use motivations, this form of recreational use relates mostly to the enhancement and social motives. A substantial minority broke with this trivializing view, however, and regarded cannabis as a proper entheogen, although perhaps not of the same stature as the classical psychedelics. Such spiritual use connects primarily to what Simons et al. (1998) called the expansion motive, although there are clearly aspects of both enhancement and social motives in this form of use as well, since these spiritually motivated cannabis users reported experiences with significantly higher levels of love and an improved connection with other people than non-spiritually motivated users. This result is congruent with previous findings on entheogenic spirituality (Johnstad 2018). Among both types of cannabis users, furthermore, one third of the respondents reported a coping motive for use, as they endorsed using cannabis because they wanted to forget or escape from personal problems.

In interviews, spiritually motivated cannabis users often reported having meditative or introspective cannabis sessions, while recreationally motivated users did not report such an introspective focus. This finding is congruent with the advice from Gray (2017) that cannabis experiences will be more powerful when the user engages with the experience in inner silence. In the survey data, furthermore, there were clear correlations between having a spiritual motivation for cannabis use and ending up with spiritual-type cannabis experiences. Convergent findings thus support the hypothesis that users’ approach to cannabis in terms of motivation and usage pattern has considerable impact upon their experiences.

Participants in interviews often emphasized their intention of maintaining a moderate usage frequency of cannabis in order to preserve its spiritual value, or, in some cases, acknowledged that over-frequent use had somewhat diminished their cannabis experiences. Interviewees generally found that because of a build-up of tolerance, overuse of entheogens would entail a loss of effect, and it seems likely that habitual cannabis users experience cannabis as relatively mild because of such tolerance. This finding is congruent with research that has obtained evidence of tolerance to the subjective intoxication effects of cannabis (Colizzi and Bhattacharyya 2018; Gorelick et al. 2013). The median number of use occasions over the last 12 months in the survey data was not significantly different for spiritually motivated users and recreational users, however, and more frequent cannabis users reported more positive cannabis experiences and indicated that their use had better long-term consequences. These findings agree with Bresin and Mekawi’s (2019) meta-analysis of the relations between cannabis use motives and outcomes, where expansion and enhancement motives were associated with a higher frequency of use. The present study thus identified an inconsistency between the interview and survey data, as the emphasis on moderation among some interviewees was not reflected in a lower frequency of use among spiritually motivated cannabis users in the survey. One interpretation of this finding is that while a build-up of tolerance to the subjective effects of cannabis reduces the intensity of the spiritual experience, users often choose to go for frequent low-intensity experiences instead of infrequent high-intensity experiences. This interpretation is congruent with a dynamic identified in interviews, where overuse led to experiences that were less powerful, but still regarded as spiritually relevant.

Both interview and survey respondents reported that cannabis and psychedelics use had an overall positive impact on physical and psychological health, personal happiness, sociability, and spiritual practice. Spiritually motivated users reported significantly better consequences for psychological health and spiritual practice, while users with an escapist motivation reported significantly worse consequences for psychological health, sociability, and personal happiness. These findings generally agree with Bresin and Mekawi’s (2019) meta-analysis, although their analysis only tested for negative outcomes. They found that coping motives predicted both a higher cannabis frequency and more problematic use, while expansion and enhancement motives predicted a higher frequency of use but not problematic use.

The main limitations of this explorative study were that participants were recruited via online psychedelic communities, and had to self-select for participation. It has previously been found that participants recruited on the Internet have more education and higher incomes (Hamilton and Bowers 2006), which might potentially bias findings. While the Internet is probably more accessible to those with lower education and income levels today than it was in 2006, the Internet recruitment in this study may have served to exclude some cannabis and psychedelics users. Survey participants who completed the survey had higher education and higher scores on the personality traits Openness and Conscientiousness than participants who dropped out along the way, which indicates that the survey may have been received more positively by respondents with more education and specific personality structures. Furthermore, the study recruited mainly among current users of cannabis and psychedelics, who as a group are probably favorably inclined towards such drug use. The study should therefore be considered biased towards positive results.

The study suggests several directions for future research. In the survey sample of psychedelics users, 25% endorsed having a spiritual motivation for cannabis use and reported cannabis experiences that, at least in some respects, resembled experiences with psychedelics. It would be interesting to know the extent of spiritual cannabis use among other samples of cannabis users: is this a widespread or a marginal social phenomenon? It is possible that the psychedelics users recruited for this study are more spiritually inclined than nonusers are and that selection bias has affected present findings, but it is also possible that a substantial proportion of the general cannabis-using population would endorse having a spiritual motivation for use, if only someone asked them about this. Furthermore, if some form for entheogenic spirituality based on cannabis use is widespread in Western societies, we should know more about its characteristics. The question of how usage frequency impacts on the intensity and meaningfulness of spiritual cannabis experience via a tolerance effect also deserves further investigation, for instance via a retrospective study that asks participants to rate and compare past and present experiences.

## Conclusions

The majority of cannabis users in this study regarded cannabis as a recreational drug devoid of entheogenic features. A minority of the sample endorsed having a spiritual motivation for cannabis use and regarded it as an important entheogen, although not necessarily as efficacious in this regard as the classical psychedelics. Such spiritual users differed from recreational users both in their mode of engagement with cannabis and in the type of experiences obtained. Recent research has not given much attention to spiritual aspects of cannabis use, but the study indicates that spiritually motivated use remains prevalent and deserves further study.

## Supplementary information

**Additional file 1.** Supplemental online material: Survey questionnaire as PDF, SPSS dataset, and online appendix.

## Data Availability

The dataset generated or analyzed during this study is included in this published article [and its supplementary information files].

## Abbreviations

2C-B: 2,5-dimethoxy-4-bromophenethylamine
5-MeO-DMT: 5-methoxy-*N,N*-dimethyltryptamine
DMT: *N,N*-Dimethyltryptamine
EMCDDA: European Monitoring Centre for Drugs and Drug Addiction
LSD: Lysergic acid diethylamide
MDMA: 3,4-Methylene-dioxy​methamphetamine
NSSDS: Norwegian Social Science Data Services
UNODC: United Nations Office on Drugs and Crime
