# Supplementary material for: Cannabis as entheogen: survey and interview data on the spiritual use of cannabis
Source: J Cannabis Res. 2020 Sep 22;2:30. doi: 10.1186/s42238-020-00032-2 (PMC7819316; doi:10.1186/s42238-020-00032-2)
Supplement: Supplementary file 1 — Additional file 1. Supplemental online material: Survey questionnaire as PDF, SPSS dataset, and online appendix. [file 42238_2020_32_MOESM1_ESM.zip › Cannabis and Psychedelics User Survey.pdf]

Cannabis and Psychedelics User Survey

This survey is part of a project at the Institute of Sociology at the University of Bergen. To participate, you must be 18 years or older, have a good understanding of English, and have at least some experience with psychedelics. Participation is fully anonymous: neither your IP address nor other identifying data is recorded. Please only answer the survey once.

I am 18 years or older

- ☐ Yes
- ☐ No

I understand English well

- ☐ Yes
- ☐ No

Demographics

Age

- ☐ 18-19
- ☐ 20-29
- ☐ 30-39
- ☐ 40-49
- ☐ 50-59
- ☐ 60+

Gender

- ☐ Female
- ☐ Male
- ☐ Other

Education

- ☐ Have not completed high school
- ☐ High school
- ☐ Some university
- ☐ Bachelor's degree
- ☐ Master's degree
- ☐ PhD

Occupation. Check all that apply.

- [ ] Full-time job
- [ ] Student
- [ ] Part-time job
- [ ] Unemployed
- [ ] Pensioner
- [ ] Other

Relationship status

- [ ] Single
- [ ] Partner
- [ ] Married
- [ ] Widow/widower

Number of children

- [ ] 0
- [ ] 1
- [ ] 2
- [ ] 3+

Geographical location at the present time

- [ ] Africa
- [ ] America (North)
- [ ] America (South)
- [ ] Asia
- [ ] Europe (East)
- [ ] Europe (West)
- [ ] Middle East
- [ ] Oceania

Spiritual or religious background. Check all that apply.

- [ ] Hindu
- [ ] Christian
- [ ] Jewish
- [ ] Secular/Humanist
- [ ] Muslim
- [ ] Buddhist
- [ ] New Age/Alternative
- [ ] Other

Personality

I see myself as...

	Disagree strongly	Disagree moderately	Neither agree nor disagree	Agree moderately	Agree strongly
Critical, quarrelsome	❑	❑	❑	❑	❑
Anxious, easily upset	❑	❑	❑	❑	❑
Extraverted, enthusiastic	❑	❑	❑	❑	❑
Reserved, quiet	❑	❑	❑	❑	❑
Disorganized, careless	❑	❑	❑	❑	❑
Open to new experiences, complex	❑	❑	❑	❑	❑
Sympathetic, warm	❑	❑	❑	❑	❑
Dependable, self-disciplined	❑	❑	❑	❑	❑
Calm, emotionally stable	❑	❑	❑	❑	❑
Conventional, uncreative	❑	❑	❑	❑	❑

How often have you taken risks of the following kinds

	Never	Rarely	Sometimes	Often	Very often
Financial risks (e.g. gambling, risky investments)	❑	❑	❑	❑	❑
Social risks (e.g. taking an unpopular stance on a controversial issue, publicly challenging a rule or decision)	❑	❑	❑	❑	❑
Recreational risks (e.g., rock-climbing, scuba diving)	❑	❑	❑	❑	❑
Safety risks (e.g. fast driving, cycling without a helmet)	❑	❑	❑	❑	❑
Career risks (e.g. quitting a job without another to go to)	❑	❑	❑	❑	❑

Spirituality and religion

Which spiritual or religious traditions do you currently feel connected to? Check all that apply.

- ❑ Hinduism
- ❑ Buddhism
- ❑ Secular/Humanist
- ❑ New Age/Alternative
- ❑ Judaism
- ❑ Islam
- ❑ Christianity
- ❑ Other

Do you do any regular spiritual or self-developmental practice? Check all that apply.

- ❑ Chanting/Singing
- ❑ Dream work
- ❑ Energy work
- ❑ Hypnosis/Regression
- ❑ Mantra
- ❑ Meditation
- ❑ Prayer
- ❑ Reading spiritual or religious texts

- Visualization/Inner journeys
- Yoga (hatha yoga)
- Other
- None

Non-psychedelic drug use

Which of the following drugs do you currently use?

	Daily	A few times per week	A few times per month	A few times per year	Never
Ketamine					
Alcohol					
Cocaine					
Other plant-based relaxants or stimulants (Betel, Kava, Kratom etc.)					
Nootropics					
Amphetamines					
Cigarettes or tobacco					
Opiates and opiate-like products					
GHB					
Coffe or tea					

Cannabis

Do you have experience with the use of cannabis?

- Yes
- No

Cannabis usage pattern

For how many years have you used (or did you use) cannabis?

- Less than a year
- 1-3 years
- 3-5 years
- 5-10 years
- More than 10 years

How many times have you used cannabis?

- Once
- 2-3 times
- 4-6 times
- 7-10 times
- 11-20 times

- 21-50 times
- 51-100 times
- 100+ times

How often have you used cannabis over the last 12 months?

- Not at all
- Once
- 2-3 times
- 4-6 times
- 7-10 times
- 11-20 times
- 21-50 times
- 51-100 times
- 100+ times

What is (or was) your most common social environment for using cannabis?

- Alone
- With a single partner
- With a small group of close friends
- With a group of friends and acquaintances
- At a party, night club, concert, festival or other public event

How far in advance do (or did) you usually plan the use of cannabis? Choose nearest option.

- One day or less in advance
- A few days in advance
- A few weeks in advance
- A few months in advance
- A year in advance

Motivation for cannabis use

What were your original motivations for starting to use cannabis? Check all that apply.

- Adventure
- Ego death experience
- Curiosity
- Spiritual experience
- To forget or escape from personal problems
- Socializing
- Insight and understanding for personal growth
- Psychological self-exploration
- Fun/party/recreation

- To cure or heal personal problems
- To cure or heal medical conditions
- Other

What were your motivations for continuing to use cannabis after the first period of experimentation? Check all that apply.

- Ego death experience
- To forget or escape from personal problems
- Psychological self-exploration
- Fun/party/recreation
- Curiosity
- Insight and understanding for personal growth
- Spiritual experience
- To cure or heal medical conditions
- Adventure
- Socializing
- To cure or heal personal problems
- Other

Cannabis: most meaningful experience

Consider your most meaningful cannabis experience. Which of the following emotional characteristics apply to this experience? Check all that apply.

- Peace
- Sadness
- Love
- Joy
- Disgust
- Fear
- Surprise
- Anger or hate
- Other

Consider your most meaningful cannabis experience. Which of the following cognitive characteristics apply to this experience? Check all that apply.

- Inner visions
- Insight into the world
- Words cannot describe the experience
- Insight into yourself
- Confusion
- Ego death or dissolution
- Insight into other people and your relations with them

- Other

Consider your most meaningful cannabis experience. Which of the following relational characteristics apply to this experience? Check all that apply.

- Contact with transcendent forces
- Violent behavior
- Unity with transcendent forces
- Feeling of homecoming or return to your true essence
- Improved connection with other people
- Contact with non-ordinary beings
- Regrettable behavior towards others
- Feeling of isolation from other people
- Improved connection with nature
- Other

Consider your most meaningful cannabis experience. How meaningful was this experience to you?

- Most meaningful experience of your life
- Among the five most meaningful experiences of your life
- Among the ten most meaningful experiences of your life
- The most meaningful experience of a year
- The most meaningful experience of a month
- An everyday experience

Cannabis: typical experience

Consider a typical cannabis experience. Which of the following emotional characteristics apply to this experience? Check all that apply.

- Disgust
- Fear
- Joy
- Sadness
- Anger or hate
- Peace
- Love
- Surprise
- Other

Consider a typical cannabis experience. Which of the following cognitive characteristics apply to this experience? Check all that apply.

- Inner visions
- Confusion
- Insight into other people and your relations with them

- Words cannot describe the experience
- Insight into yourself
- Ego death or dissolution
- Insight into the world
- Other

Consider a typical cannabis experience. Which of the following relational characteristics apply to this experience? Check all that apply.

- Violent behavior
- Contact with non-ordinary beings
- Feeling of homecoming or return to your true essence
- Contact with transcendent forces
- Improved connection with other people
- Improved connection with nature
- Regrettable behavior towards others
- Feeling of isolation from other people
- Unity with transcendent forces
- Other

Cannabis: worst experience

If you only have good experiences with cannabis, then your worst experience is simply your least good experience. In other words, your worst experience is not necessarily a bad experience.

Consider your worst cannabis experience. Which of the following emotional characteristics apply to this experience? Check all that apply.

- Peace
- Disgust
- Anger or hate
- Fear
- Joy
- Love
- Surprise
- Sadness
- Other

Consider your worst cannabis experience. Which of the following cognitive characteristics apply to this experience? Check all that apply.

- Insight into yourself
- Insight into other people and your relations with them
- Inner visions
- Ego death or dissolution
- Insight into the world
- Confusion

- Words cannot describe the experience
- Other

Consider your worst cannabis experience. Which of the following relational characteristics apply to this experience? Check all that apply.

- Contact with transcendent forces
- Regrettable behavior towards others
- Contact with non-ordinary beings
- Feeling of isolation from other people
- Unity with transcendent forces
- Improved connection with other people
- Violent behavior
- Improved connection with nature
- Feeling of homecoming or return to your true essence
- Other

Consider your worst cannabis experience. How difficult was this experience to you?

- Most difficult experience of your life
- Among the five most difficult experiences of your life
- Among the ten most difficult experiences of your life
- The most difficult experience of a year
- The most difficult experience of a month
- An everyday experience / not difficult

Consider your worst cannabis experience. How would you judge the long-term consequences of this particular experience?

- Long-term negative impact on life and health
- Mostly negative long-term consequences for life and health
- No significant or mixed long-term consequences for life and health
- Mostly positive long-term consequences for life and health
- Long-term positive impact on life and health

Cannabis: consequences of use

What are the long-term consequences of using cannabis on your physical health?

- Serious worsening of overall health
- Moderate worsening of overall health
- No significant consequences for health
- Moderate improvement of overall health
- Serious improvement of overall health

What are the long-term consequences of using cannabis on your

psychological health?

- Serious worsening of overall health
- Moderate worsening of overall health
- No significant consequences for health
- Moderate improvement of overall health
- Serious improvement of overall health

What are the long-term consequences of using cannabis for your personal happiness?

- Much less happy
- Less happy
- Same as before
- More happy
- Much more happy

What are the long-term consequences of using cannabis for your ability to get along with other people?

- Much reduced relationship ability
- Reduced relationship ability
- Same as before
- Improved relationship ability
- Much improved relationship ability

What are the long-term consequences of using cannabis for your spiritual practice?

- Much reduced intensity of practice
- Reduced intensity of practice
- Same as before
- Improved intensity of practice
- Much improved intensity of practice

Have you had flashback experiences because of your cannabis use?

- No
- Yes, and they were mainly positive experiences
- Yes, and they were mainly negative experiences

Psychedelics survey

How many times have you used these psychedelics?

	Never	Once	2-3 times	4-6 times	7-10 times	11-20 times	21-50 times	51-100 times	100+ times
LSD	☐	☐	☐	☐	☐	☐	☐	☐	☐
MDMA	☐	☐	☐	☐	☐	☐	☐	☐	☐

Psilocybin/Magic mushrooms	☐	☐	☐	☐	☐	☐	☐	☐	☐
Ayahuasca (or analogues)	☐	☐	☐	☐	☐	☐	☐	☐	☐
DMT (smoked)	☐	☐	☐	☐	☐	☐	☐	☐	☐
5-MeO-DMT	☐	☐	☐	☐	☐	☐	☐	☐	☐
Mescaline/Peyote	☐	☐	☐	☐	☐	☐	☐	☐	☐
Salvia divinorum	☐	☐	☐	☐	☐	☐	☐	☐	☐
2C family (2C-B etc.)	☐	☐	☐	☐	☐	☐	☐	☐	☐

You will now be asked about your experiences with psychedelics. Please choose one of the psychedelics from the list below. All the remaining questions in this survey will be about your experience with this one psychedelic.

Please choose one psychedelic that you have experience with and wish to speak about in this survey:

☐ Salvia divinorum
☐ Psilocybin/Magic mushrooms
☐ 5-MeO-DMT
☐ LSD
☐ MDMA
☐ Ayahuasca (or analogues)
☐ 2C family (2C-B etc.)
☐ Mescaline/Peyote
☐ DMT (smoked)

Usage pattern

Note: all questions pertain to the psychedelic you chose previously.

For how many years have you used (or did you use) this psychedelic?

☐ Less than a year
☐ 1-3 years
☐ 3-5 years
☐ 5-10 years
☐ More than 10 years

How many times have you used this psychedelic?

☐ Once
☐ 2-3 times
☐ 4-6 times
☐ 7-10 times
☐ 11-20 times
☐ 21-50 times

- 51-100 times
- 100+ times

How often have you used this psychedelic over the last 12 months?

- Not at all
- Once
- 2-3 times
- 4-6 times
- 7-10 times
- 11-20 times
- 21-50 times
- 51-100 times
- 100+ times

What is (or was) your most common social environment for using this psychedelic?

- Alone
- With a single partner
- With a small group of close friends
- With a group of friends and acquaintances
- At a party, night club, concert, festival or other public event

How far in advance do (or did) you usually plan the use of this psychedelic? Choose nearest option.

- One day or less in advance
- A few days in advance
- A few weeks in advance
- A few months in advance
- A year in advance

Motivation for psychedelic use

Note: all questions pertain to the psychedelic you chose previously.

What were your original motivations for starting to use this psychedelic? Check all that apply.

- To forget or escape from personal problems
- Insight and understanding for personal growth
- Psychological self-exploration
- Curiosity
- To cure or heal medical conditions
- Spiritual experience
- Socializing
- To cure or heal personal problems

- Ego death experience
- Adventure
- Fun/party/recreation
- Other

What were your motivations for continuing to use this psychedelic after the first period of experimentation? Check all that apply.

- To cure or heal personal problems
- Socializing
- Insight and understanding for personal growth
- To cure or heal medical conditions
- Fun/party/recreation
- Spiritual experience
- Ego death experience
- Curiosity
- To forget or escape from personal problems
- Adventure
- Psychological self-exploration
- Other

Most meaningful experience

Note: all questions pertain to the psychedelic you chose previously.

Consider your most meaningful experience with this psychedelic. Which of the following emotional characteristics apply to this experience? Check all that apply.

- Sadness
- Surprise
- Fear
- Disgust
- Anger or hate
- Joy
- Peace
- Love
- Other

Consider your most meaningful experience with this psychedelic. Which of the following cognitive characteristics apply to this experience? Check all that apply.

- Insight into yourself
- Insight into the world
- Words cannot describe the experience
- Inner visions
- Confusion

- Insight into other people and your relations with them
- Ego death or dissolution
- Other

Consider your most meaningful experience with this psychedelic. Which of the following relational characteristics apply to this experience? Check all that apply.

- Regrettable behavior towards others
- Contact with non-ordinary beings
- Violent behavior
- Improved connection with other people
- Improved connection with nature
- Unity with transcendent forces
- Feeling of homecoming or return to your true essence
- Contact with transcendent forces
- Feeling of isolation from other people
- Other

Consider your most meaningful experience with this psychedelic. How meaningful was this experience to you?

- Most meaningful experience of your life
- Among the five most meaningful experiences of your life
- Among the ten most meaningful experiences of your life
- The most meaningful experience of a year
- The most meaningful experience of a month
- An everyday experience

Typical experience

Note: all questions pertain to the psychedelic you chose previously.

Consider a typical experience with this psychedelic. Which of the following emotional characteristics apply to this experience? Check all that apply.

- Surprise
- Joy
- Anger or hate
- Fear
- Peace
- Sadness
- Love
- Disgust
- Other

Consider a typical experience with this psychedelic. Which of the following cognitive characteristics apply to this experience? Check all that apply.

- Insight into the world
- Ego death or dissolution
- Insight into other people and your relations with them
- Insight into yourself
- Inner visions
- Confusion
- Words cannot describe the experience
- Other

Consider a typical experience with this psychedelic. Which of the following relational characteristics apply to this experience? Check all that apply.

- Feeling of homecoming or return to your true essence
- Regrettable behavior towards others
- Improved connection with nature
- Contact with non-ordinary beings
- Improved connection with other people
- Unity with transcendent forces
- Violent behavior
- Contact with transcendent forces
- Feeling of isolation from other people
- Other

Worst experience

Note: all questions pertain to the psychedelic you chose previously.

If you only have good experiences with this psychedelic, then your worst experience is simply your least good experience. In other words, your worst experience is not necessarily a bad experience.

Consider your worst experience with this psychedelic. Which of the following emotional characteristics apply to this experience? Check all that apply.

- Sadness
- Love
- Peace
- Fear
- Joy
- Anger or hate
- Surprise
- Disgust
- Other

Consider your worst experience with this psychedelic. Which of the following cognitive characteristics apply to this experience? Check all that

apply.

- Confusion
- Insight into yourself
- Insight into other people and your relations with them
- Words cannot describe the experience
- Ego death or dissolution
- Inner visions
- Insight into the world
- Other

Consider your worst experience with this psychedelic. Which of the following relational characteristics apply to this experience? Check all that apply.

- Improved connection with nature
- Feeling of isolation from other people
- Feeling of homecoming or return to your true essence
- Regrettable behavior towards others
- Contact with non-ordinary beings
- Unity with transcendent forces
- Violent behavior
- Improved connection with other people
- Contact with transcendent forces
- Other

Consider your worst experience with this psychedelic. How difficult was this experience to you?

- Most difficult experience of your life
- Among the five most difficult experiences of your life
- Among the ten most difficult experiences of your life
- The most difficult experience of a year
- The most difficult experience of a month
- An everyday experience / not difficult

Consider your worst experience with this psychedelic. How would you judge the long-term consequences of this particular experience?

- Long-term negative impact on life and health
- Mostly negative long-term consequences for life and health
- No significant or mixed long-term consequences for life and health
- Mostly positive long-term consequences for life and health
- Long-term positive impact on life and health

Consequences of use

Note: all questions pertain to the psychedelic you chose previously.

What are the long-term consequences of using this psychedelic on your physical health?

- Serious worsening of overall health
- Moderate worsening of overall health
- No significant consequences for health
- Moderate improvement of overall health
- Serious improvement of overall health

What are the long-term consequences of using this psychedelic on your psychological health?

- Serious worsening of overall health
- Moderate worsening of overall health
- No significant consequences for health
- Moderate improvement of overall health
- Serious improvement of overall health

What are the long-term consequences of using this psychedelic for your personal happiness?

- Much less happy
- Less happy
- Same as before
- More happy
- Much more happy

What are the long-term consequences of using this psychedelic for your ability to get along with other people?

- Much reduced relationship ability
- Reduced relationship ability
- Same as before
- Improved relationship ability
- Much improved relationship ability

What are the long-term consequences of using this psychedelic for your spiritual practice?

- Much reduced intensity of practice
- Reduced intensity of practice
- Same as before
- Improved intensity of practice
- Much improved intensity of practice

Have you had flashback experiences because of your use of this psychedelic?

- No
- Yes, and they were mainly positive experiences
- Yes, and they were mainly negative experiences

Thank you!

Your responses have been recorded anonymously, and will contribute to a deeper understanding of psychedelics use and its consequences.
