# Supplementary material for: Cannabis as entheogen: survey and interview data on the spiritual use of cannabis
Source: J Cannabis Res. 2020 Sep 22;2:30. doi: 10.1186/s42238-020-00032-2 (PMC7819316; doi:10.1186/s42238-020-00032-2)
Supplement: Supplementary file 1 — Additional file 1. Supplemental online material: Survey questionnaire as PDF, SPSS dataset, and online appendix. [file 42238_2020_32_MOESM1_ESM.zip › Online appendix - Cannabis as entheogen.docx]

**Cannabis as an entheogen: Survey and interview data on the spiritual use of cannabis**

Online appendix.

| **Table A. Differences between participants who completed the full survey (*N* = 213) and participants who opted out along the way (*N* = 106).** | | | |
| --- | --- | --- | --- |
| Participants who completed the full study: | *t* | *df* | *p* |
| were more likely to be a pensioner | 3.06 | 212 | .003 |
| had higher scores on the personality trait openness | 2.68 | 109 | .009 |
| had higher scores on the personality trait conscientiousness | 2.00 | 287 | .047 |
| were more likely to report a connection to Buddhism | 2.34 | 154 | .021 |
| were more likely to report a connection to Hinduism | 2.01 | 184 | .046 |
| had lower present use of alcohol | 2.22 | 277 | .028 |
| had higher present use of ketamine | 2.22 | 176 | .027 |
| had higher present use of nootropics | 2.20 | 185 | .029 |
| had higher present use of the 2C family of psychedelics | 2.37 | 42 | .022 |
| had higher present use of MDMA | 2.65 | 235 | .009 |
| had higher present use of psilocybin | 2.79 | 235 | .006 |
| reported more joy in a typical cannabis experience | 2.07 | 248 | .040 |
| reported more peace in a typical cannabis experience | 3.15 | 58 | .003 |
| reported more insight into the world in a typical cannabis experience | 2.25 | 71 | .027 |
| reported more contact with non-ordinary beings in a typical cannabis experience | 2.88 | 204 | .004 |
| reported more connection with nature in a typical cannabis experience | 3.46 | 70 | .001 |
| Note: *t* = value on the independent t-test. *df* = degrees of freedom. *p* = significance level. | | | |
